# Reduced diversity of intestinal T-cell receptor repertoire in patients with Crohn’s disease

**DOI:** 10.3389/fcimb.2022.932373

**Published:** 2022-08-10

**Authors:** Sung Noh Hong, Joo-Young Park, So-Yun Yang, Chansu Lee, Young-Ho Kim, Je-Gun Joung

**Affiliations:** ^1^ Department of Medicine, Samsung Medical Center, Sungkyunkwan University School of Medicine, Seoul, South Korea; ^2^ Stem Cell & Regenerative Medicine Center, Samsung Medical Center, Seoul, South Korea; ^3^ Samsung Genome Institute, Samsung Medical Center, Seoul, South Korea; ^4^ Department of Biomedical Science, College of Life Science, CHA University, Seongnam, South Korea

**Keywords:** Crohn’s disease, T-cell receptor, T-cell receptor repertoire, RNA sequencing, clonotype

## Abstract

**Background:**

The intestinal microenvironment directly determines the human T-cell receptor (TCR) repertoire. Despite its extreme diversity, TCR repertoire analysis may provide a better understanding of the immune system in patients with inflammatory bowel disease.

**Methods:**

To investigate TCR repertoires in the intestinal mucosa, RNA sequencing was performed for inflamed and non-inflamed intestinal mucosa samples obtained from 13 patients with Crohn’s disease (CD) and healthy mucosa from nine non-IBD controls.

**Results:**

The gene expression frequency of the TCR repertoire showed a clear separation between inflamed mucosa of patients with CD and healthy mucosa of non-IBD controls in the hierarchical clustering heatmap. The richness of TCR repertoires measured by the Chao1 index did not show a significant difference among groups, whereas diversity measured by the D50 diversity index was decreased in the inflamed mucosa of CD patients. Rare/small TCR clonotypes occupied a large proportion of TCR repertoires in healthy mucosa of controls, whereas expanded clonotypes were common in inflamed mucosa of patients with CD. Segment usages of TRAV2, TRAV22, TRAV40, TRJ14, TRAJ51, TRBV1, TRBV21.1, and TRBJ1.5 were significantly decreased in CD patients. KEGG enrichment analysis identified the enrichment of several KEGG pathways, including inflammatory bowel disease (*p* = 0.0012), Th1 and Th2 cell differentiation (*p* = 0.0011), and intestinal immune network for IgA production (*p* = 0.0468).

**Conclusions:**

The diversity of the TCR repertoire is reduced in inflamed mucosa of CD patients, which might contribute to intestinal inflammation.

## Introduction

Host T-cell responses to commensal bacteria are fairly consistent in the steady state. However, they can become altered and exaggerated in an inflamed intestinal mucosa in inflammatory bowel disease (IBD). A diverse repertoire of T cells monitors intestinal microbial diversity and prevents the expansion of potential pathogens (Alcover et al., 2018). In the pathogenesis of Crohn’s disease (CD), T cells play a pivotal role in mounting altered and excessive immune responses against intestinal luminal antigens that might otherwise be tolerated under homeostatic conditions. Conventional αβ T cells—the majority of T cells in the gut—encounter cognate antigens and proliferate to produce clones of T-cell receptor (TCR)-identical cells which defend against the intrusion of potential pathogens (Doorenspleet et al., 2017). The characteristics of intestinal microbiota in patients with IBD include reduced microbial diversity and proliferation of potential pathogens (Ni et al., 2017). When the intestinal mucosal immune system is continuously exposed to intestinal microbiota, the TCR repertoire in patients with IBD is thought to be distorted due to responses to altered microbial populations. Oligoclonal T-cell expansion has been observed in murine IBD models (Matsuda et al., 2000; Probert et al., 2007) and in blood and inflamed mucosa of IBD patients (Probert et al., 2007; Camus et al., 2014).

Therefore, TCR repertoire analysis may help us gain a better understanding of immune system features and disease etiology in patients with IBD, in particular those with unknown antigenic triggers. However, the extreme diversity of the TCR repertoire represents a major analytical challenge (Rosati et al., 2017). Traditionally, the TCR repertoire has been analyzed using fragment length measures of the complementarity-determining region (CDR)3 motif by spectratyping (Fozza et al., 2017; Holtmeier et al., 2002). The expansion of specific CDR3 clonotypes has been identified (Probert et al., 1996). Recently, next-generation sequencing-based technologies are most widely employed for high-throughput analysis of the immune cell repertoire (Rosati et al., 2017). Significant alterations in the TCR repertoire have been demonstrated in cancer, autoimmune diseases, and infectious diseases (Gros et al., 2014; Han et al., 2014; Muraro et al., 2014; Bai et al., 2015; Saravanarajan et al., 2020). However, few studies have explored the TCR repertoire in patients with CD. Here, we applied an RNA sequencing-based approach to investigate differences in T-cell repertoires in the ileum of treatment-naïve patients with CD and non-IBD controls.

## Materials and methods

### Samples

All patients and controls were recruited from Samsung Medical Center after a written informed consent was obtained. The Institutional Ethical Committee of the Samsung Medical Center approved the study protocol (IRB No. 2016-02-022).

We prospectively enrolled 13 patients with active CD (mean age: 30.9 ± 10.9 years; 9 were men) who underwent diagnostic or surveillance colonoscopy (**Table 1**). CD was diagnosed and classified based on established diagnostic guidelines for CD (Ye et al., 2009). Clinical characteristics of the enrolled patients were as follows: Montreal classification A1/A2/A3, n = 2/n = 8/n = 3; L1/L2/L3, n = 6/n = 2/n = 5; B1/B2/B3, n = 5/n = 5/n = 3. During colonoscopy, two to four biopsy samples were taken from active ulcer margins (inflamed intestinal mucosa, n = 13) and endoscopically normal-appearing mucosa (non-inflamed intestinal mucosa, n = 13) from each patient with CD. Biopsy specimens were immediately frozen in liquid nitrogen and stored at −80°C.

**Table 1:** Characteristics of enrolled patients with Crohn’s disease and non-IBD controls.

		Crohn’s disease (n = 13)	Non-IBD controls (n = 9)
Gender, n (%)			
	Male	9 (69)	5 (56)
	Female	4 (31)	4 (44)
Age (years), mean ± S.D.		30.9 ± 10.9	51.8 ± 15.5
Non/ex-smoker/current smoker		12 (92)/1 (8)	3 (33)
Montreal classification, n (%)			
	Age at diagnosis, A1/A2/A3	2 (15)/8 (62)/3 (23)	–
	Location, L1/L2/L3/L4	6 (46)/2 (15)/5 (39)/0 (0)	–
	Perianal modifier	8 (62)	–
	Behavior, B1/B2/B3	5 (39)/5 (39)/3 (23)	–
Biopsy site for tissue acquisition			
Inflamed mucosa			
Ileum		8 (62)	–
Colon		5 (39)	–
Non-inflamed mucosa			
Ileum		4 (31)	–
Colon		9 (69)	9 (100)
Crohn’s Disease Activity Index (CDAI) at tissue acquisition, n (%)			
Inactive (CDAI <150)		4 (31)	–
Active (CDAI ≥150)		9 (31)	–
Medication at tissue acquisition, n (%)			
	None	7 (54)	–
	Yes	6 (46)	–
	Corticosteroid	2	–
	Azathioprine	4	–
	Anti-TNF-α antibody	2	–
Activity at 1 year after enrollment, n (%)			
	Inactive (CDAI <150)	9 (69)	–
	Active (CDAI ≥150)	4 (31)	–

As controls, nine non-IBD patients were enrolled prospectively (mean age: 49.7 ± 14.9 years; 6 were men) who underwent colonoscopies for colorectal polyp removal. Endoscopically normal-appearing healthy colonic mucosa specimens were obtained from the ascending colon of each non-IBD control (healthy mucosa of non-IBD control, n = 9). They were immediately frozen in liquid nitrogen and stored at −80°C.

### RNA sequencing

Total RNA was extracted by combining two to four biopsy samples obtained using an RNeasy Mini Kit (Qiagen, Valencia, CA, USA) according to the manufacturer’s instructions. Library construction was performed using a TruSeq RNA Sample Prep Kit v.2 (Illumina, San Diego, California, USA) and 500 ng of genomic RNA extracted from each sample. Briefly, in order to obtain a paired-end RNA sequencing library, several steps including a reverse transcription reaction with poly-(dT) primers using SuperScript™ II reverse transcriptase (Invitrogen/Life Technologies, Grand Island, NY, USA), cDNA amplification, end-repair, 3′-end adenylation, adapter ligation, and amplification were conducted. The quantity and quality of the library were measured using a Qubit 2.0 Fluorometer (Thermo Fisher Scientific, Waltham, MA, USA) and a 4200 TapeStation instrument (Agilent Technologies, Santa Clara, CA, USA). Sequencing of the transcriptome library was carried out using the 100-bp paired-end mode of a TruSeq Rapid PE Cluster kit and a TruSeq Rapid SBS kit (Illumina) on a HiSeq 2500 sequencing platform (Illumina).

### TCR analysis

In the preprocessing step, in order to identify TCR clonotypes of each sample, next-generation sequencing (NGS) data of raw reads were aligned against the international ImMunoGeneTics Library (https://github.com/repseqio/library-imgt/releases/tag/v5) using the MiXCR v.3.0.10 tool (https://github.com/milaboratory/mixcr). Sequencing reads were aligned to reference V, D, J, and C regions of the T-cell receptor α chain (TRA) and β chain (TRB). Clonotypes were then assembled using aligned reads to extract CDR3 regions. In the post-processing step, VDJtools (https://github.com/mikessh/vdjtools) and “immunarch” R package (https://immunarch.com/) were used to analyze the number of clonotypes, clonotype proportion, and repertoire diversity. These tools were also utilized to display a segment usage heatmap. Sequence logos of CDR3 DNA and amino acid sequences were visualized using WebLogo (http://weblogo.berkeley.edu/logo.cgi).

Sequence reads as FASTQ files were mapped against the human reference genome build 19 (hg19) using TopHat v.2.0.6 (http://ccb.jhu.edu/software/tophat/index.shtml). Raw read counts mapped to genes were measured using the BAM format file with HTSeq version 0.6.1 (https://htseq.readthedocs.io). Differentially expressed genes were identified using the DESeq R package (www.huber.embl.de/users/anders/DESeq/). Enriched pathways were identified using the DAVID database (https://david.ncifcrf.gov/). Gene set enrichment analysis was conducted with the negatively and positively correlated genes with the usage of TCR segments, and the enriched KEGG pathway terms were identified.

## Results

### Expression pattern of TCR repertoire genes in the intestinal mucosa of Crohn’s disease and non-IBD controls

**Figure 1:**
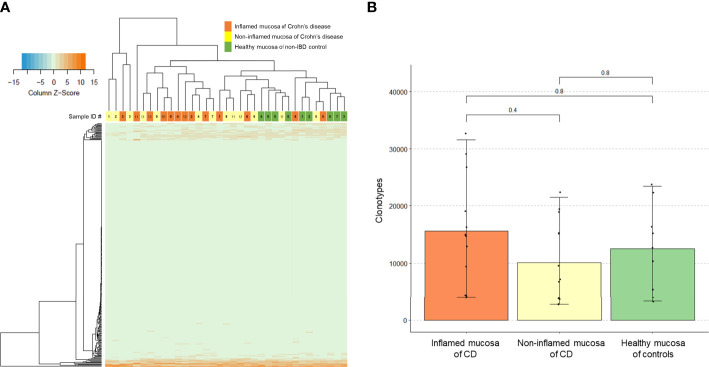
T-cell receptor (TCR) clonotype count. **(A)** Expression of TCR repertoire genes and **(B)** counts of unique TCR clonotypes in the inflamed mucosa and non-inflamed mucosa of Crohn’s disease (CD) patients and healthy mucosa of non-inflammatory bowel disease (IBD) controls.

Expression profiles of the intestinal TCR repertoires exhibited a clear separation in the hierarchical clustering heatmap between the inflamed mucosa in patients with CD and healthy mucosa of non-IBD controls (**Figure 1A**). Some non-inflamed mucosa of CD patients were clustered with the inflamed mucosa of CD patients while others were clustered with the healthy mucosa of controls. However, the number of unique clonotypes in the inflamed mucosa of CD patients did not differ from that in the healthy mucosa of controls (**Figure 1B**).

**Figure 2:**
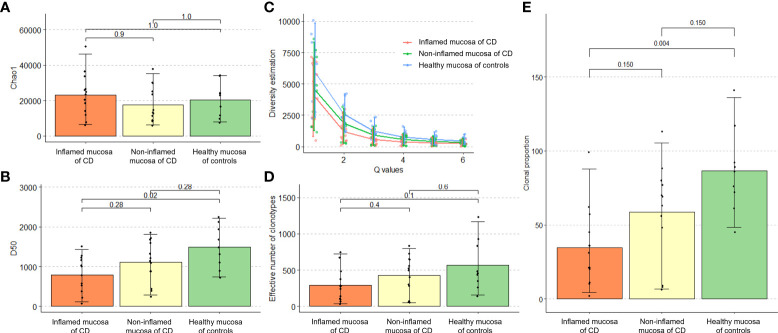
Richness and diversity of TCR repertoire in patients with Crohn’s disease and non-IBD controls. **(A)** Alpha diversity measured by the Chao1 richness estimator. **(B)** Hill number diversity index. **(C)** D50 diversity index. **(D)** True diversity index. **(E)** Number of clonotypes occupying 10% of the TCR repertoire.

TCR repertoire richness (the number of unique TCR sequences) measured using Chao1 estimates did not differ between the groups (**Figure 2A**). There was no significant difference in Hill numbers between the groups, although such number was numerically lower in the inflamed mucosa of CD patients (**Figure 2B**). On the other hand, TCR diversity estimation using the D50 diversity index, a recently developed immune diversity estimate, and the number of clonotypes occupying 50% of TCR repertoires were significantly lower in the inflamed mucosa of CD patients than in healthy mucosa of non-IBD controls (*P* = 0.02, **Figure 2C**). The true diversity index showed no significant difference between the groups, although it was numerically lower in the inflamed mucosa of CD patients (**Figure 2D**). The number of clonotypes occupying 10% of repertoires was significantly lower in the inflamed mucosa of CD patients than in non-IBD controls (*P* = 0.004). The number in the non-inflamed mucosa of patients with CD tended to be low compared with controls and high compared with inflamed mucosa of CD patients (**Figure 2E**). These results indicated that the richness of clonotypes did not differ, whereas the diversity of TCR repertoires might be reduced in the inflamed mucosa of CD patients compared with that in healthy mucosa of non-IBD controls.

### Distribution of clonotypes occupying TCR repertoires in the intestinal mucosa of Crohn’s disease and non-IBD controls

**Figure 3:**
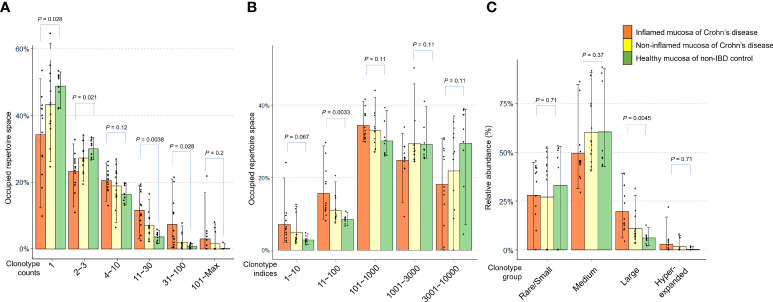
Distribution of clonotypes occupying TCR repertoires in inflamed and non-inflamed mucosa of patients with Crohn’s disease and healthy mucosa of non-IBD controls. **(A)** Proportion of TCR repertoire occupied according to clonotype counts. **(B)** Proportion of TCR repertoire occupied according to clonotype indices. **(C)** Relative abundance of rare/small, medium, large, and hyperexpanded clonotype.

Next, we evaluated the occupied proportion in the TCR repertoire according to the clonotypes classified by clonotype counts in patients with CD and non-IBD controls. The expression frequency of clonotypes determines the count distribution (Puelma Touzel et al., 2020). Low clonotype counts indicate rare clonotypes. The clonotype count 1 and 2~3 occupied more repertoire space in the controls than in CD patients. In the mucosa of CD, the TCR repertoire space occupied by rare clonotypes was higher in inflamed mucosa than in non-inflamed mucosa (*P* = 0.028 and *P* = 0.021, respectively). On the other hand, high clonotype counts indicating expanded clonotypes occupied more repertoire space in CD patients than in controls (clonotype count 11~30: *P* = 0.0038 and clonotype count 31~100: *P* = 0.028, respectively) (**Figure 3A**). The clonotype index is ordered by decreasing clonotype size. A low clonotype index indicates an expanded clonotype whereas a high clonotype index indicates a rare/small clonotype group. The clonotype indices 11~100 in the inflamed mucosa of CD patients occupied more repertoire space than the non-IBD controls (*P* = 0.0033, **Figure 3B**). When the clonotype was classified into rare/small (0 < log expression level ≤ 1e-04), medium (1e-04 < log expression level ≤ 0.001), large (0.001 < log expression level ≤ 0.01), and hyperexpanded (0.01 < log expression level ≤ 1), the relative abundance of large clonotype was significantly higher in the inflamed mucosa of CD patients than in the healthy mucosa of controls (*P* = 0.0045, **Figure 3C**). These data suggest that rare and small clonotypes might be more common in the healthy mucosa of controls, whereas expanded (large and hyperexpanded) clonotypes might occupy a large proportion of the TCR repertoire in the inflamed mucosa of CD patients.

### Expression pattern of TCRα and TCRβ gene segments

**Figure 4:**
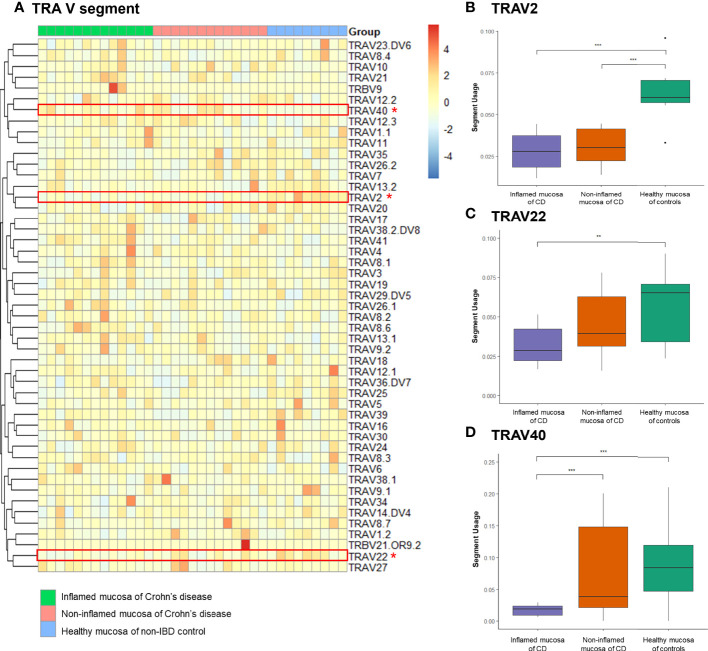
Expression frequency of TCRα-variable (TRAV) segments. **(A)** Heatmap. Red dot and line indicate differentially expressed TRAV segments. **(B)** TRAV2 segment usage, **(C)** TRAV2 segment usage, and **(D)** TRAV40 segment usage. *p < 0.05, **p < 0.01, and ***p < 0.001.

Expression frequencies of the TCRα variable (TRAV) and junctional (TRAJ) segments and the TCRβ variable (TRBV) and junctional (TRBJ) segments were evaluated. A heatmap illustrated the expression profile of TRAV segments in the inflamed and non-inflamed mucosa of CD and healthy mucosa of controls (**Figure 4A**). Among the TRAV clonotypes, the expression of TRAV2 segment was significantly reduced in the inflamed and non-inflamed mucosa of CD patients compared to that in the healthy mucosa of controls (**Figure 4B**). The usage of TRAV2 and TRAV40 segments was significantly decreased in the inflamed mucosa of CD patients compared to that in the healthy mucosa of controls (**Figures 4C, D**).

**Figure 5:**
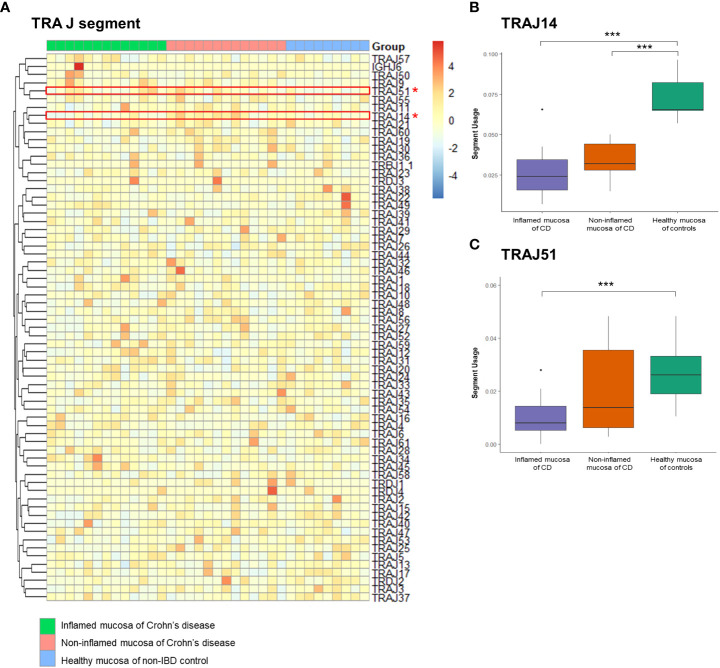
Expression frequency of TCRα junctional (TRAJ) segments. **(A)** Heatmap. Red dot and line indicate differentially expressed TRAJ segments. **(B)** TRAV2 segment usage and **(C)** TRAJ51 segment usage. *p < 0.05, and ***p < 0.001.

A heatmap illustrated the expression profile of TRAJ segments (**Figure 5A**). Among the TRAJ clonotypes, the expression of the TRAJ14 segment was significantly reduced in the inflamed mucosa of CD patients compared to that in the non-inflamed mucosa of CD patients and the healthy mucosa of controls (**Figure 5B**). TRAJ51 segment usage was significantly reduced in the inflamed mucosa of CD patients compared to that in the healthy mucosa of controls (**Figure 5C**).

**Figure 6:**
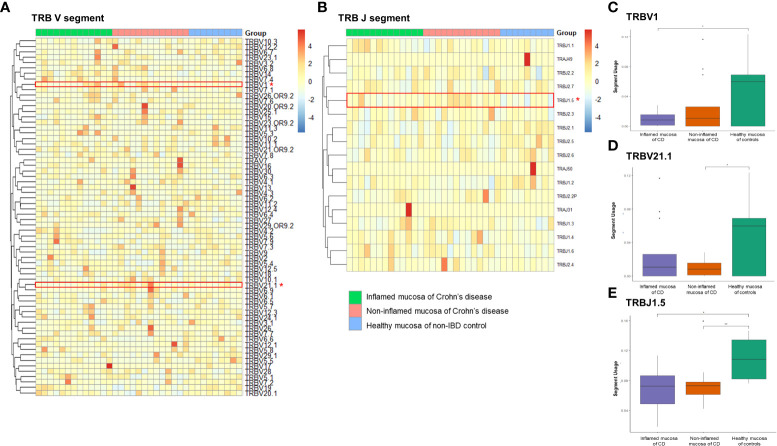
Expression frequency of TCRβ variable (TRBV) and junctional (TRBJ) segments. Heatmap for **(A)** TRBV and **(B)** TRABJ segment expression. Red dot and line indicate differentially expressed TRAJ segments. **(C)** TRBV1 segment usage, **(D)** TRBV21.1 segment usage, and **(E)** TRAV2 segment usage. *p < 0.05 and **p < 0.01.

Expression frequencies of the TRBV and TRBJ segments were illustrated with a heatmap (**Figures 6A, B**). Among the TRB clonotypes, the expression of the TRBV1 segment was significantly reduced in the inflamed mucosa of CD patients compared to that in the healthy mucosa of controls (**Figure 6C**). The expression of the TRBV21.1 segment was significantly reduced in the non-inflamed mucosa of CD patients compared to that in the healthy mucosa of controls (**Figure 6D**). TRBJ1.5 segment usage was significantly reduced in the inflamed and non-inflamed mucosa of CD patients compared to that in the healthy mucosa of controls (**Figure 6E**).

**Figure 7:**
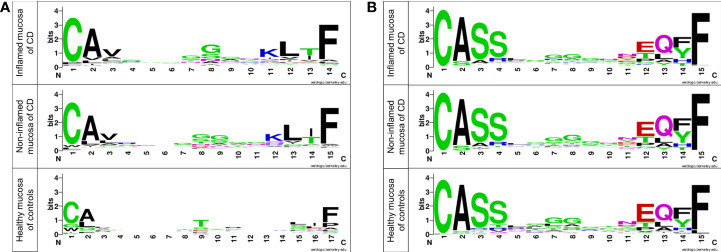
Sequence logos of amino acids in the CDR3 motif of **(A)** TRA and **(B)** TRB in patients with CD and controls. Each logo consists of stacks of symbols, one stack for each position in the sequence. The overall height of the stack indicates the sequence conservation at that position, while the height of symbols within the stack indicates the relative frequency of each amino at that position (Crooks et al., 2004).

Different T-cell clones have different CDR3 sequences (Yang et al., 2018). The amino acid residues in the CDR3 motif of TRA and TRB were evaluated and illustrated using sequence logos as described in a previous study (Crooks et al., 2004). Several positions of the CDR3 motif of TRA showed differences between patients with CD and non-IBD controls. They were similar between inflamed and non-inflamed mucosa of CD patients (**Figure 7A**). Nonetheless, amino acid sequences did not show strong differences in the CDR3 motif of TRB between the inflamed and non-inflamed mucosa in patients with CD or between the mucosa of CD patients and controls (**Figure 7B**).

### Gene set enrichment analysis with correlated genes with TCR segment usages

**Figure 8:**
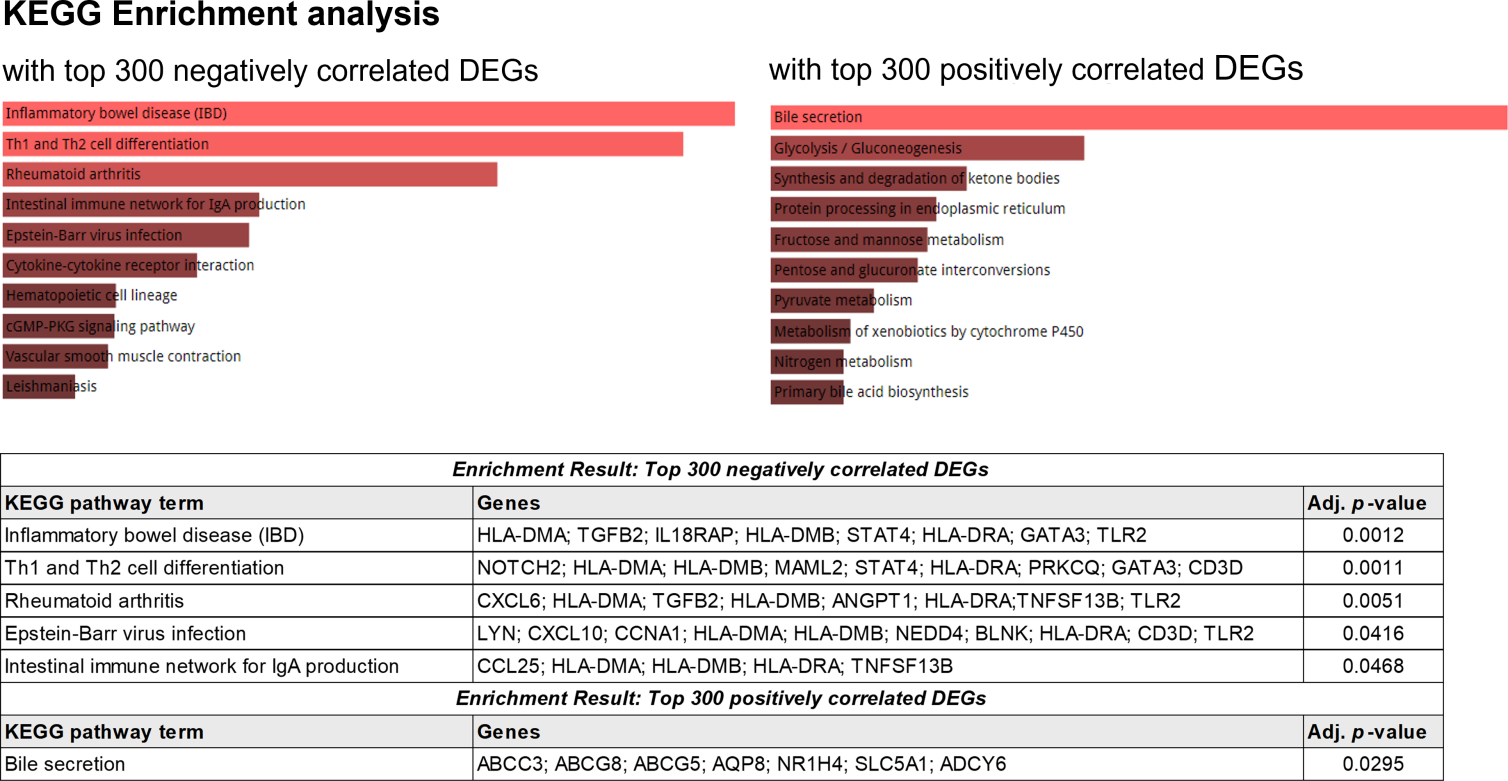
KEGG enrichment analysis using the top 300 genes showing positive correlations with the frequency of TCR.

Next, gene set enrichment analysis (GSEA) was performed with negatively and positively correlated genes with the usage of TCR segments (**Figure 8**). The enrichment result with top 300 genes, which showed a negative correlation with the usage of TCR segments, was identified through enrichment of several KEGG pathway terms, including inflammatory bowel disease (adjusted *p* = 0.0012), Th1- and Th2-cell differentiation (adjusted *p* = 0.0011), rheumatoid arthritis (adjusted *p* = 0.0051), Epstein–Barr virus infection (adjusted *p* = 0.0416), and intestinal immune network for IgA production (adjusted *p* = 0.0468). The enrichment result with top 300 genes, which had a positive correlation with the usage of TCR segments, was associated with the enrichment of bile secretion (adjusted *p* = 0.0295).

## Discussion

The intestinal microbiota plays a critical role in the training and development of major components of the host innate and adaptive immune systems (Lee and Mazmanian, 2010; Zheng et al., 2020). Growing evidence demonstrates that microbiota–immunity interactions are imbalanced under defined environmental contexts. Such imbalance is believed to contribute to the pathogenesis of IBD. Whether the host can distinguish pathogenic from symbiotic bacteria may affect the spectrum of host–microbiota relationships, resulting in mutualism and autoimmunity (Lee and Mazmanian, 2010; Lee et al., 2019; Lloyd-Price et al., 2019; Saravanarajan et al., 2020). T cells can recognize an immense range of pathogens by utilizing the versatility of the TCR clonotype and initiate specific adaptive immune responses (Wang et al., 2007). The clonotype is a molecular description of the unique sequences required to produce the antigen specificity of TCRs (Izraelson et al., 2018). The sum of all TCR clonotypes of one individual is termed the TCR repertoire or TCR profile (Rosati et al., 2017). This study demonstrated that the expression pattern of the TCR repertoire differs between the intestinal mucosa in patients with CD and non-IBD controls.

In patients with CD, microbial diversity was decreased. Relative proportions of *Proteobacteria* and *Fusobacteria* were increased with concomitant decreases in relative abundances of *Firmicutes* and *Bacteroidetes* (Manichanh et al., 2006; Mukhopadhya et al., 2012; Eun et al., 2016). It would have been great to obtain microbiome data of enrolled patients, but that was not possible in this study. Nevertheless, we found that the diversity of the TCR repertoire measured by the D50 diversity index, the true diversity index, and Hill numbers gradually decreased in the inflamed mucosa of patients with CD, the non-inflamed mucosa of patients with CD, and the healthy mucosa of non-IBD controls. Statistically significant differences of diversity indices between the inflamed mucosa of patients with CD and the non-IBD controls were noticed. The proportion of TCR clonotypes occupying the TCR repertoires was altered in inflamed mucosa of patients with CD. Rare clonotypes were popular in healthy mucosa of controls, whereas expanded clonotypes occupied a major proportion of the TCR repertoire in the inflamed mucosa of patients with CD. We then performed a GSEA with a gene set correlated with the TCR repertoire and identified the association with the IBD-related signaling pathway. Taken together, these results indicate that alterations to the TCR repertoire in inflamed mucosa patients with CD contribute to active intestinal inflammation.

In previous studies, TCR repertoires in IBD patients were analyzed by next-generation sequencing using blood or intestinal biopsy samples. In a Japanese study, intestinal lamina propria mononuclear cells and peripheral blood mononuclear cells (PBMCs) were isolated from surgical specimens of 12 patients with IBD (including 7 with CD) and compared them with PBMCs from 10 healthy controls (Kakuta et al., 2020). RNA-sequencing of TCR transcripts identified reduced diversity of TCRα and TCRβ in PBMCs, consistent with our result. TCR diversity in PBMCs was significantly lower in CD than in ulcerative colitis (UC). An Irish study has compared TCR repertoires of PBMCs and inflamed intestines collected from 11 patients with IBD (including 7 patients with CD). Intestinal TCR repertoires exhibited lower clonotype diversity and greater clonal expansion than those in PBMCs (Saravanarajan et al., 2020). Differential usages of TRAV12–3, TRAJ37, and TRAJ43 in inflamed intestine were identified. However, TRAV2, TRAV22, TRAV40, TRJ14, TRAJ51, TRBV1, TRBV21.1, and TRBJ1.5 identified in our study were not included in that Irish study. A Dutch study has collected inflamed and non-inflamed intestinal samples of 19 patients with CD before and after 8 weeks of biologic therapy (Doorenspleet et al., 2017). Only the TRB repertoire was analyzed. It found that expanded clonotypes and the largest clonotypes occupied as much as 58% of the TCR repertoire, similar to our results in that expanded clonotypes occupied a large proportion of the TCR repertoire. Chapman et al. have obtained biopsies from the neo-ileum of 17 patients undergoing evaluation for postoperative recurrence of CD and compared them with normal terminal ileums of four non-IBD controls (Chapman et al., 2016). They found that TCR diversity in intestinal mucosa was significantly lower than that of matched PBMCs, indicating the expansion of certain T-cell populations in inflamed intestinal tissues. A French study has analyzed 57 patients with CD who have undergone a surgery (Allez et al., 2019). They found that smoking was associated with reduced TCR repertoire diversity and induced clonal expansions. Patients with an increased proportion of T-cell clonal expansion at the time of surgery showed an increased risk of postoperative endoscopic recurrence. However, previous studies could not demonstrate a direct relationship between CD-associated molecular signaling and differential gene expression of TCRα and TCRβ chains. The present study further performed GSEA with the gene set correlated with the usage of TCR segments. KEGG mapping identified the enriched IBD, Th1 and Th2 cell differentiation, and intestinal immune network for IgA production.

In addition, each TCR chain contains three hypervariable loops in its structure, termed CDR1–3. CDR1 and CDR2 encoded by TCR germline variable (V) gene regions are well conserved across different TRAV and TRBV subfamilies (Arden, 1998). CDR3 is encoded by the junctional region between the V and J genes, which is the region of the TCR in direct contact with peptide antigens. It determines TCR specificity (Rosati et al., 2017). The limited diversity shown by CDR1 and CDR2 contrasts with the extreme CDR3 junctional diversity. Therefore, CDR3 is often used as a region of interest to determine T-cell clonotypes as it is highly unlikely that two T cells will express the same CDR3 nucleotide sequence unless they are derived from the same clonally expanded T cell (Turner et al., 2006; Miles et al., 2011). In our study, the nucleotide sequences and specific amino acid sequences of the CDR3 motif of TRA showed differences between patients with CD and non-IBD controls. This supports the hypothesis that expanded T-cell clones may differ between patients with CD and controls.

Previous studies have shown that T-cell repertoires may be maintained stable in the intestines of CD patients regardless of inflamed and uninflamed mucosa (Camus et al., 2014; Doorenspleet et al., 2017). However, treatment for CD can affect the TCR repertoire. In a Dutch study, biologics treatment induced a loss of some of the expanded TCR clonotypes in biologics responders compared with patients who did not respond to therapy (Doorenspleet et al., 2017). In the present study, the TCR repertoire showed a difference between inflamed and non-inflamed mucosa of CD patients, although the difference did not reach statistical significance. In particular, the usage of the TRAV40 segment was increased significantly in non-inflamed mucosa compared with that in the inflamed mucosa of CD patients. The sampling of inflamed areas and concomitant therapy including biologics can affect the expansion of clonotypes. In the present study, the mucosal samples were obtained from the inflamed and non-inflamed areas of the same patients, which is a strength to evaluate the TCR repertoire in patients with CD. Biopsy samples of histology for the inflamed mucosa of CD had moderate to severe activity, whereas those for non-inflamed mucosa had a mild activity. The histology of biopsy samples for the healthy mucosa of non-IBD controls showed no pathologic alterations.

Our study has some limitations. First, the small sample size was a major limitation, which might have resulted in the result that diversity indices of the non-inflamed mucosa of patients with CD could not show clear differences compared with the inflamed mucosa of patients with CD and healthy mucosa of controls. Second, we collected paired samples from the inflamed and non-inflamed mucosa from the same patients. The pairing of inflamed and non-inflamed tissue extended from the ileum to the colon. Doorenspleet et al. reported that the intestinal TCR repertoire of patients with CD is remarkably stable between the ileum and colon. We focused on TCR repertoires of the inflammation site regardless of whether the samples were from the colon or ileum. However, the difference in T-cell population could affect the results. In addition, paired analysis of inflamed and non-inflamed samples obtained from the same patient was not performed. Third, in contrast to lower microbial richness in patients with IBD (Alam et al., 2020), the richness of TCR repertoires was not significantly different in this study. Differences of richness in TCR repertoires and clonotypes should be reevaluated using a large number of IBD cohort samples. In addition, the transcriptome of unfractionated biopsies contains different cell types. Repertoire richness contraction was less pronounced for memory CD4+, CD8+ T cells, and Treg cells (Qi et al., 2014). As mucosal αβ T cells are predominantly CD8+ in the epithelium and mostly CD4+ in the lamina propria, the diversity of the TCR repertoire might have been affected by the CD4:CD8 ratio and the proportion of Treg in the endoscopic biopsy samples. However, these could not be evaluated in our study. In the aspect of the methodology, the latest RNA-sequencing technique such as single-cell RNA-sequencing can provide detailed individual cell level data (Rosati et al., 2017), which is more appropriate to identify the alteration of the TCR repertoire in patients with CD. In a subsequent study, with a large number of samples obtained from CD patients with different phenotypes, single-cell RNA-sequencing should be performed methodologically.

## Conclusions

In the intestinal mucosa of CD patients, the expanded clonotypes occupied a large proportion of the TCR repertoire, which induced a reduced diversity of the TCR repertoire. This phenomenon is more pronounced in inflamed mucosa than in non-inflamed mucosa of CD compared to the healthy mucosa of non-IBD controls. The usage of the TRAV2 and TRBJ1.5 segments was significantly decreased in the mucosa of CD patients regardless of the presence of inflammation. Compared to the healthy mucosa of controls, usages of TRAV2, TRAV40, TRAJ14, TRAJ51, and TRBV1 segments were significantly decreased in the inflamed mucosa of CD patients. The altered TCR gene expression might have contributed to the activation of the inflammatory signaling pathway in the intestine in patients with CD. Considering the reduction in microbial diversity and the expansion of specific pathogens in patients with CD, the change in TCR repertoires identified in this study may be the corresponding result of alterations to the intestinal microbiota. Further research should be needed to confirm the correlation of the alteration of TCR repertoires with diagnosis, phenotypes, prognosis, and drug responses in patients with CD. In addition, future studies with a larger sample are required to explore the characteristics of clonal T-cell expansions and the identification of the antigens that trigger these responses. For this purpose, it will be necessary to perform the multi-omics analysis with combination of the TCR repertoire and intestinal microbial metagenomic data. This may could shed light on the unveiled pathophysiology of CD and lead to the development of a novel targeted therapy.

## Data availability statement

The datasets presented in this study can be found in GEO (accession number, GSE208303).

## Ethics statement

The study protocol involving human participants were reviewed and approved by The Institutional Ethical Committee of the Samsung Medical Center (IRB No. 2016-02-022). The study participants/patients provided written informed consent. Ethical review and approval was not required for the animal study.

## Author contributions

Conceptualization, J-GJ, SNH. Methodology, J-GJ. Formal analysis, J-YP, S-YY, J-GJ. Investigation, J-YP, S-YY, J-GJ. Resources, SNH, CL. Data curation, J-YP, S-YY, J-GJ. Writing—original draft preparation, SNH. Writing—review and editing, SNH, J-GJ. Visualization, SNH, J-YP, S-YY, J-GJ. Supervision, SNH, CL, Y-HK, J-GJ. Funding acquisition, SNH, J-GJ. All authors have read and agreed to the published version of the manuscript.

## Funding

This work was supported by the National Research Foundation of Korea (NRF) funded by the Korean government (MSIP) (2019R1A2C2010404, 2019R1I1A01062205, and 2019R1F1A1061365), Future Medicine 20*30 Project of the Samsung Medical Center, and an intramural grant from the CHA University (202100330001).

## Conflict of interest

The authors declare that the research was conducted in the absence of any commercial or financial relationships that could be construed as a potential conflict of interest.

## Publisher’s note

All claims expressed in this article are solely those of the authors and do not necessarily represent those of their affiliated organizations, or those of the publisher, the editors and the reviewers. Any product that may be evaluated in this article, or claim that may be made by its manufacturer, is not guaranteed or endorsed by the publisher.

## Abbreviations

TCR, T-cell receptor; CD, Crohn’s disease; IBD, inflammatory bowel disease; NGS, next-generation sequencing; TRA, T-cell receptor α chain; TRB, T-cell receptor β chain; TRAV, TCRα variable; TRAJ, TCRα junctional; TRBV, TCRβ variable; TRBJ, TCRβ junctional; GSEA, Gene set enrichment analysis; PBMCs, peripheral blood mononuclear cells; CDR, complementarity-determining region.
